# Empirical evidence of nonlinearity in bottom up effect in a marine predator–prey system

**DOI:** 10.1098/rsbl.2022.0309

**Published:** 2022-11-02

**Authors:** Joël M. Durant, Kotaro Ono, Øystein Langangen

**Affiliations:** ^1^ Centre for Ecological and Evolutionary Synthesis (CEES), Department of Biosciences, University of Oslo, PO Box 1066 Blindern, NO-0316 Oslo, Norway; ^2^ Section for Aquatic Biology and Toxicology (AQUA), Department of Biosciences, University of Oslo, PO Box 1066 Blindern, NO-0316 Oslo, Norway; ^3^ Institute for Marine Research (IMR), PO Box 1870 Nordnes, Bergen 5817, Norway

**Keywords:** Bayesian statistic, Gompertz model, species competition, predation, threshold modelling

## Abstract

The strength of species interactions may have profound effects on population dynamics. Empirical estimates of interaction strength are often based on the assumption that the interaction strengths are constant. Barents Sea (BS) cod and capelin are two fish populations for which such an interaction has been acknowledged and used, under the assumption of constant interaction strength, when studying their population dynamics. However, species interactions can often be nonlinear in marine ecosystems and might profoundly change our understanding of food chains. Analysing long-term time series data comprising a survey over 37 years in the Arcto-boreal BS, using a state-space modelling framework, we demonstrate that the effect of capelin on cod is not linear but shifts depending on capelin abundance: while capelin is beneficial for cod populations at high abundance; below the threshold, it becomes less important for cod. Our analysis therefore shows the importance of investigating nonlinearity in species interactions and may contribute to an improved understanding on species assemblages.

## 1. Introduction

Climate change is profoundly affecting and altering marine systems [1]. Indirect effects of climate change, such as alteration of species interactions, might have a stronger impact on population dynamics than the direct warming effects [2,3]. The environment can also have a non-additive effect (e.g. threshold) on population dynamics in terrestrial [4,5] and marine [6–10] systems alike resulting in different population equilibrium and dynamics [4]. Marine systems are prone to nonlinear transitions under climate warming [1] and overfishing [11] that may also lead to altered population dynamics [12,13]. A prime example of such nonlinear transition is the Atlantic cod [10,11,14]. However, such nonlinear transitions have seldom been studied in relation to species interactions (but see [12]). To study such interactions, Wootton & Emmerson [15] suggest the use of long-term time series to take into account nonlinearity and process errors. This can be achieved using state-space modelling approaches [12,16] in data rich systems such as the Barents Sea (BS) [17].

Here, we explore the population dynamics of two interacting species: BS capelin *Mallotus villosus* and Northeast Arctic (NEA) cod *Gadus morhua*. Both species are known to interact in the BS and affect each other's population [18]. Indeed, predation by NEA cod on BS capelin is thought to have delayed the capelin stock's recovery after its collapses [13]. In addition, BS capelin is considered to be the main food for NEA cod [19,20] and low capelin stock was blamed for the very low cod catches at the end of 1980s [21]. Both species population dynamics are well documented to be affected by environmental variables (e.g. [22,23]).

Here, we applied a Gompertz state-space model [12,16] on long-term time series data comprising a survey over 37 years of BS capelin and NEA cod [24] aiming to (i) assess whether there is a linear or nonlinear interaction between cod and capelin, and (ii) understand what a nonlinear dynamics means for the population and the trophic interactions in the system.

## 2. Methods

**Figure 1.**
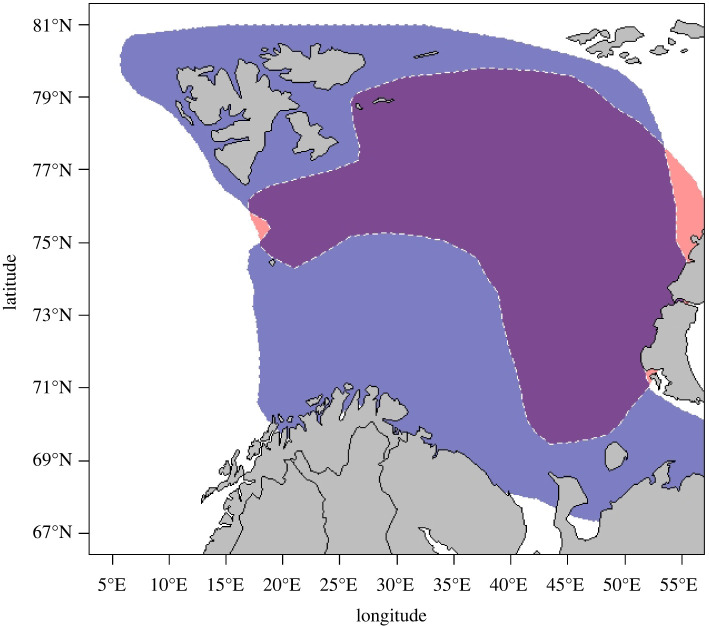
Approximate feeding distributions in the Barents Sea of the Northeast Arctic cod (blue) and the capelin (red). The map is redrawn from Bakketeig *et al*. [25].

**Figure 2.**
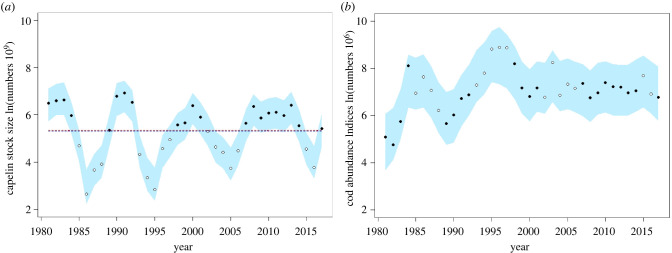
Posterior predictive check on abundances indices. The dots show the acoustic (for capelin in *a*) and trawl (for cod in *b*) survey log-transformed abundance estimates (i.e. data) and the blue bands are the 95% predictive intervals. For both plots, filled data points are for the years with capelin stock size over the estimated threshold between > 5.30 (blue dotted line in figure *a*) and ≤ 5.34 billions (red dotted line in figure *a*).

We analysed jointly the change in population abundance for the BS capelin and NEA cod from the BS (figure 1). Population data (1981–2019) were published fish stock assessments data (table 9.4 for the capelin, table A3 for the cod) [24]. Capelin stock size in numbers are estimates from the August–September acoustic survey, and cod abundance are indices in numbers from the January–March bottom trawl surveys in the BS (figure 2).

In addition, we used two climatic variables (the Kola transect sea temperature, ST, and the winter North Atlantic Oscillation, wNAO) as potential environmental drivers of capelin and cod population dynamics (e.g. [22,23]). The sea temperature (1921–2019) is an aggregated average over the upper 200 m at five stations (3 to 7) on the Kola meridian transect (33°30' E, 70°30'–72°30' N) in the BS (http://www.pinro.ru/; [26]). The December to March North Atlantic Oscillation index [27] represents North Atlantic-scale climate effects (1964–2019). Both ST and NAO were standardized to *z*-scores.

### (a) Model description

The analyses were based on a Gompertz state-space model [12] reparameterized as in Stenseth *et al*. [4] incorporating competition (intra- and interspecific, respectively *a_i_*_,*i*_ (with the intra-specific interaction set to 1 [4,28]) and *a_i_*_,*j*_) and environmental variables (*a_i_*_,st_ and *a_i_*_,nao_) effects. The model (table 1: equation (1)) incorporated also a Gaussian distributed stochastic term (*ε*) to acknowledge our inadequate understanding of the complexity of the dynamics of population *i* (i.e. the process error: equation (2)).

**Table 1.** Summary of the equations. With *N_i_*_,yr_ the abundance for the species *i* at year *yr*. Process error *ε_i_*_,yr_ of the population *i* with variance $\sigma_{i,\mathrm{proc}}^{2}$. Observation error ln(Obs*_i_*_,yr_) of the population *i* with variance $\sigma_{i,\mathrm{obs}}^{2}$. ST, sea temperature; wNAO, winter North Atlantic Oscillation; *a* or *b*, parameters with the first subscript the modelled species and the second subscript the variable—environment or other species—affecting this species.

formulation	equation
$\ln(N_{i,\mathrm{yr}})=a_{i,0}+a_{i,i}\cdot \ln(N_{i,\mathrm{yr}-1})+a_{i,j}\cdot \ln(N_{\;j,\mathrm{yr}-1})+a_{i,\mathrm{st}}\cdot ST_{\mathrm{yr}-1}+a_{i,\mathrm{nao}}\cdot \mathrm{wNA}O_{\mathrm{yr}-1}+\varepsilon_{i,\mathrm{yr}}$	1
$\varepsilon_{i,\mathrm{yr}}∼\mathrm{normal}(0,\;\sigma_{i,\mathrm{proc}}^{2})$	2
$\ln(N_{i,\mathrm{yr}})=a_{i,0}+a_{i,i}\cdot \ln(N_{i,\mathrm{yr}-1})+a_{i,j}\cdot \ln(N_{\;j,\mathrm{yr}})+a_{i,\mathrm{st}}\cdot ST_{\mathrm{yr}-1}+a_{i,\mathrm{nao}}\cdot \mathrm{wNA}O_{\mathrm{yr}-1}+\varepsilon_{i,\mathrm{yr}}$	3
$\ln({\mathrm{Obs}}_{i,\mathrm{yr}})∼\mathrm{normal}(\ln(N_{i,\mathrm{yr}}),\;\sigma_{i,\mathrm{obs}}^{2})$	4
$\ln(N_{i,\mathrm{yr}})=\{\begin{array}{c} a_{i,0}+a_{i,i}\cdot \ln(N_{i,\mathrm{yr}-1})+a_{i,j}\cdot \ln(N_{\;j,\mathrm{yr}-1})+a_{i,\mathrm{st}}\cdot ST_{\mathrm{yr}-1}+a_{i,\mathrm{nao}}\cdot \mathrm{wNA}O_{\mathrm{yr}-1}+\varepsilon_{i,\mathrm{yr}},\mathrm{if}\;\ln(N_{\;j,\mathrm{yr}-1})<\theta \;\\ b_{i,0}+a_{i,i}\cdot \ln(N_{i,\mathrm{yr}-1})+b_{i,j}\cdot \ln(N_{\;j,\mathrm{yr}-1})+a_{i,\mathrm{st}}\cdot ST_{\mathrm{yr}-1}+a_{i,\mathrm{nao}}\cdot \mathrm{wNA}O_{\mathrm{yr}-1}+\varepsilon_{i,\mathrm{yr}},\;\mathrm{otherwise}\; \end{array}$	5

Since the sampling of capelin population is in August–September while in January–March for the cod, the cod survey at year_yr_ was conducted between the capelin surveys at year_yr−1_ and year_yr_. We took this into account when modelling the capelin by using the cod abundance estimate at year_yr_ (*N_j_*_,yr_) instead of year_yr−1_ (*N_j_*_,yr−1_) as described in equation (3).

We assumed that the observed abundances (Obs; from trawl survey for cod and acoustic survey for capelin) were normally distributed (in log scale) with variance term *σ*^2^*_i_*_,obs_ around the true log population values for the species *i* (equation (4)). Prior specifications are found in the accompanying codes in the electronic supplementary material.

To detect possible nonlinear dynamics, we tested for potential pairwise interactions between all explanatory variables (table 1) using Bürmann's expansion [29]. In short, Bürmann's expansion test checks interaction between pairs of variables by analysing the residuals between additive models with or without interaction thus finding the best fit and reports significance. Only when nonlinearity was detected did we include a threshold non-additive effect in the Gompertz state-space model [12]. In our case (see results), the threshold non-additive effect let the growth potential of species *i* and the effect of species *j* on species *i* (*a_i_*_,0_/*b_j0_* and *a_i_*_,*j*_ */b_ij_*, respectively) change according to whether the threshold variable (*X*) was below or above some threshold level *θ* (equation (5)).

To detect if and at what value the covariate *X* has a meaningful threshold effect, the model calculates the log-likelihood of the process equation (i.e. the underlying population dynamics) for each value of *X* in the data (i.e. capelin abundance ln(N_cap_), see codes in electronic supplementary material). A threshold is identified when a single value *θ* of *X* produces a large spike in log-likelihood (electronic supplementary material, figure S3). In which case, the threshold value is located somewhere between ≥*θ_b_* and <*θ* with *θ_b_* being the first value lower than the selected *θ*. Moreover, to remove any ‘border' effects i.e. spurious detection of a threshold value due to very unequal partitioning of the data (e.g. 95% below threshold versus 5% above), our model searched for a potential threshold value only within the 20–80 percentiles of the available values of *X* (21 values used out of 37).

We used a Bayesian Markov chain Monte Carlo approach to jointly estimate all parameters (for both capelin and cod) in a single model for the period 1981–2019. We used the *Stan software* via the R packages *rstan* (v. 2.21.3) and *shinystan* [30]. A likelihood function was created based on the model and data, and in combination with the prior distributions of the parameters, the posterior distributions were estimated. Weakly informative priors were used in order to let the data drive the inferences except for the process and observation error variances. The latter were not identifiable alone thus we included an informative prior on the ratio of the process to observation error variance centred around 1 (*Normal*(1, 0.5)) [31]. A sensitivity test with a ratio centred around 0.5 and 2 (respectively, *Normal*(0.5, 0.5) and *Normal*(2, 0.5)) showed that the choice of the exact value did not affect our results (electronic supplementary material, figure S1). Note that there were no indication of correlation between the estimated process errors of the two species and hence they were modelled as such (electronic supplementary material, figure S2).

We used four independent chains with 50 000 iterations each, where the first 30 000 iterations were discarded as ‘burn-in' iterations to ensure that the chains had converged. In addition, we thinned the chains with a factor 10 to reduce autocorrelation in the posterior samples and to produce a reasonable amount of output. We used the Gelman & Rubin $\hat{R}$ convergence diagnostics [32] and visual inspection of the chains to ensure convergence, and posterior predictive checks to evaluate the model fit. All analyses were conducted using the software R v. 4.1.3 [33].

## 3. Results

The Bürmann test indicated an interaction between capelin and cod abundance for both capelin and cod models (*p* < 0.05). We first used non-additive models to describe the dynamics of both species (equation (5)) but only the model for cod showed a relevant threshold (electronic supplementary material, figure S3). We then modelled capelin following equation (3) and cod following equation (5) (table 1). The cod model estimated a threshold *θ* between greater than 201 × 10^9^ and less than or equal to 209 × 10^9^ capelins (electronic supplementary material, figure S3).

Model convergence was evaluated by visual inspection of the four parallel Hamiltonian Monte Carlo chains. The chains were well mixed, had low autocorrelation after thinning and displayed no trends after the burn-in iterations. There were no divergent transitions in the chains. The Gelman & Rubin $\hat{R}$ convergence diagnostics were less than 1.002 for all model parameters thus supporting convergence. In addition, there was no systematic deviation between the fitted values and the observed time series (figure 2).

Median parameter estimates from these models are presented in table 2 (see electronic supplementary material, figure S4 for the full marginal posterior distributions). As expected, the model indicated a positive effect of the previous year abundance for both species. The environmental variables (ST and wNAO) did not show an effect for cod but ST showed an effect (positive) for capelin. For capelin, the cod showed a negative effect indicating a predation effect.

**Table 2.** Estimated parameters for the two models. Subscripts ‘cap' stands for Barents Sea (BS) capelin and ‘cod' for Northeast Arctic (NEA) cod. *θ* is the threshold value (log-transformed BS capelin abundance of 5.34 billions individuals). Note that *ca* 50% indicates that the posterior values are centred around 0. See electronic supplementary material, figure S4.

stock	parameter	median estimate	%
			>0
NEA cod	*a*_cod,0_ (cap < *θ*)	0.94	71
	*b*_cod,0_ (cap ≥ *θ*)	−1.85	28
	*a*_cod,cod_	0.84	100
	*a*_cod,cap_ (cap < *θ*)	0.03	55
	*b*_cod,cap_ (cap ≥ *θ*)	0.52	88
	*a*_cod,st_	0.06	71
	*a*_cod,nao_	0.05	81
BS capelin	*a*_cap,0_	5.74	100
	*a*_cap,cap_	0.56	100
	*a*_cap,cod_	−0.49	2
	*a*_cap,st_	0.17	86
	*a*_cap,nao_	−0.01	45

For cod, the capelin abundance, as expressed in number of capelin, has a biologically important effect. The interspecific competition term—the effect of capelin numbers on cod—was negligible (*a*_cod,cap_) when capelin was under the capelin stock size threshold and was positive (*b*_cod,cap_) over the threshold changing from 0.03 to 0.52 in a log scale (table 2; electronic supplementary material, figure S4).

This indicated that the capelin abundance had an effect on cod population only when the capelin stock was big enough (over 209 billion individuals).

## 4. Discussion

Through the use of a state-space model that combined long-term population time series with environmental variables, we illustrated how historically established species interactions may be drastically modified if explored for nonlinearity. In particular, we find empirical evidence for nonlinear change in species interaction (table 2) directly linked to prey abundance change. Non-additive population dynamics has been previously described for many species, notably for cod due to this species data availability [11,34] but seldom addressing interaction with another species [12,13].

The NEA cod is a predator of the BS capelin as shown by diet studies [19,20] and we indeed found a negative effect of cod on capelin stock, similar to previous findings [35]. Conversely, capelin abundance is expected to have a positive effect on cod stock [36,37] and our results also support the claim. However, they also indicate that the effect of capelin on cod is nonlinear and it becomes negligible for low capelin abundance.

Capelin is highly represented in the cod diet during warm years, with temperature affecting both BS capelin's distribution [38] and recruitment [35]. However, cod is a generalist predator with a diet following the community composition change [39]. Indeed, the composition of the cod diet changes over time in response to environmental conditions and the dynamics of prey populations [20,40]. This is particularly visible for the capelin proportion in the cod diet that follows the capelin population change, hence its availability as prey for the cod. This high plasticity in its diet may explain our result that cod populations are not affected by capelin abundance when the latter is under a relatively high threshold of 209 billion individuals (note that the median capelin abundance during the studied period is 227 billion individuals, data ranging from 14 billion to 1016 billion individuals). In addition, a low capelin abundance has been associated with high herring *Clupea harengus* abundance, another major predator of capelin larvae in the BS [35,41] that is also part of the cod diet [19,20].

Our model takes into account the main processes affecting the dynamics of a population i.e. interspecific competition, intra-specific competition (i.e. density dependence), and environmental conditions. However, our model does not take into account the spatial overlap of the two species that affects their interaction [42] neither the effect of the potential interaction with other species of the system (e.g. haddock *Melanogrammus aeglefinus* [12], herring [13], Polar cod *Boreogadus saida* [43]). These lacking processes are however partially taken into account by the process error in the model formulation [15] (see electronic supplementary material, figure S5).

In this study, we show that a nonlinearity in the species interactions has an impact on population dynamics and affects our understanding of the functioning of the food chain similar to what was observed for the effect of climate warming [6,12]. Stock assessment is conducted on a single species basis but increasingly incorporates some known interaction between the species of interest and climate or other species [44]. For instance, BS capelin is managed by taking into account the NEA cod predation [24]. Given the implication our results can have on the understanding of NEA cod population dynamics, our approach could be timely and necessary.

## Data Availability

Data are freely available in the report of the Arctic Fisheries Working Group (AFWG) 2019 of the International Council for the Exploration of the Sea [24] at https://ices-library.figshare.com/articles/report/Arctic_Fisheries_Working_Group_AFWG_/18618752?file=33397001; wNAO at https://climatedataguide.ucar.edu/climate-data/hurrell-north-atlantic-oscillation-nao-index-station-based. Kola sea temperature from Polar branch of the Russian Federal Institute of Fisheries and Oceanography at http://www.pinro.ru/ [45]. The data are provided in the electronic supplementary material [46].
